# Special Issue: Photoactive Materials: Synthesis, Applications and Technology

**DOI:** 10.3390/ma14030585

**Published:** 2021-01-27

**Authors:** Pierre-Alexandre Blanche

**Affiliations:** Wyant College of Optical Sciences, University of Arizona, 1630 E. University Blvd., Tucson, AZ 85721, USA; pablanche@optics.arizona.edu

The science of light–matter interaction is one of the greatest accomplishment of the past 100 years. The materials that perform useful operations by collecting light or generate light from an outside stimulus are at the center of a multitude of technologies that has permeated our daily life. Everyday we rely on quantum well laser for our telecommunication, organic light emitting diode for our displays, complementary metal–oxide–semiconductor for our camera detectors, and of course a plethora of new photovoltaic cells that harvest the sunlight to satisfy our energy need.

When editing this Special Issue of *Materials* on photoactive materials, I envision collecting articles that covers a large area from photonics, to plasmonic and holography. I wanted to showcase articles that not only demonstrated new chemical synthesis, but also gave the readers a fresh view on recent applications and technologies. I was extremely pleased by how well this call for paper was received by the community, as this Special Issue presents 11 original articles and one review, all of very high quality.

In Jung et al. [1], the authors present the synthesis of hexagonal boron nitride semiconductor doped with cerium ions. Their experiment shows how the aneling of the material impacts its fluorescence emission under UV lamp. The deep blue emission intensity was dramatically enhanced when the re-heating temperature was increased. Among other uses, this material can find application as an anti-counterfeiting ink since the marking can only be identified under UV light.

The magneto-optic effect can be used for the detection of biologic phenomena such as heart beat or brain activity. Because the field generated by these events are extremely small, synthesizing materials with a large Verdet coefficient is of major importance. This importance is demonstrated by the inclusion of three articles relevant to this topic in this Special Issue [2,3,4].

In their manuscript, Kotov et al. [2], investigated for the first time the effects of the presence of a thin protective ${\mathrm{Bi}}_{2}O_{3}$ layers on the magneto-optic properties of ultrathin highly bismuth-substituted dysprosium iron garnet layers. Their results showed a 2.7 times signal improvement with the protective oxide layer than without it.

Zhao and Li [3] took a different approach by doping sodium iron hexafluoride (${\mathrm{Na}}_{3}{\mathrm{FeF}}_{6}$) particles with monodispersed terbium ions (${\mathrm{Tb}}^{3+}$). The synthesis was obtained by a relatively easy hydrothermal process. When measuring the magnetization according to the temperature and the external magnetic field, the authors confirmed that the ${\mathrm{Na}}_{3}{\mathrm{FeF}}_{6}:\mathrm{Tb}$ particles were paramagnetic with a high magnetic moment.

By contrast, Ikesue et al. [4] investigated for the first time the magneto optical properties of a high-quality Bixbyite ceramics structure. They found out that the performances of these ceramics were far superior to those of commercial TGG (${\mathrm{Tb}}_{3}{\mathrm{Ga}}_{5}O_{12}$) crystal, which is regarded as one of the highest class of Faraday rotator material. In particular, the Verdet constant of ${\mathrm{Tb}}_{2}O_{3}$ (when x = 1.0) ceramic was the largest with value up to $154\;\mathrm{rad}\;T^{-1}\;m^{-1}$, in the wavelength range of 633 to 1064 nm. In addition, the laser damage threshold of this ceramic was $18\;J/{\mathrm{cm}}^{2}$, which is 1.8 times larger than that of TGG.

In their paper, Srinoi et al. [5], present the successful synthesis of hollow gold–silver nanoshells coated with silica shells of varying thicknesses. This was achieved by tuning the concentration of (3-aminopropyl)trimethoxysilane and sodium silicate solutions. More importantly, the authors demonstrated that none of the ${\mathrm{SiO}}_{2}$ shells had detrimental effects on the localized surface plasmon resonance peak of the gold–silver nanoshells. This result is promising for the use of that type of material in plasmon-enhanced photocatalytic applications such as water-splitting reaction to directly convert sunlight into hydrogen and oxygen gases.

In a second paper regarding the optical properties of nano-particles [6], Hurtado-Aviles et al. studied the influence of an ultrasonic stimulus on the plasmonic resonance of Au–Pt nano-particles in an ethanol suspension. The presence of the ultrasonic waves in the suspension prevented any agglomerations of the nano-particles, and it was found that the light absorption associated with surface plasmon resonance was modified by the presence of the ultrasonic wave. Ultrasound interactions together to nonlinear optical phenomena in nanofluids is a promising field for applications ranging from the modulating quantum signals to use as sensors and as acousto-optic devices.

Heavy metal elements such as lead, arsenic and mercury are highly toxic for vertebrae, including human beings. Their detection in sub ppm concentration is extremely important to guarantee food and water safety. In Kurshano et al. [7], authors are considering an optical method for detecting heavy metal ions using colloidal luminescent semiconductor quantum dots. The authors combined the magnetic properties of ${\mathrm{Fe}}_{3}O_{4}$ together with the photoluminescent properties of quantum dots of ${\mathrm{AgInS}}_{2}/\mathrm{ZnS}$ to detect metal ion concentration down to 0.01 pmm by measuring the quenching of the photoluminescence of their sensor.

Lembrikov, Ianetz, and Ben-Ezra are presenting a theoretical study of the nonlinear optical phenomena in a silicon waveguide with a smectic A liquid crystals (SLAC) core [8]. Authors have calculated the TM and TE modes in such a strongly anisotropic waveguide and have shown that the nonlinearity is related to the smectic layer normal displacement. They found that this effect is especially strong for the counter-propagating TM modes. By evaluating the pumping and signal TM mode slowly varying amplitudes and phases, they showed that the gain has a maximum value in the resonant case when the TM mode frequency difference is equal to the second sound frequency.

Scratch resistant coating would be of great advantage for optoelectronic components. In their study [9], Lazauskas et al. investigated the properties of transparent photopolymerizable thiol-ene coatings. These coatings exhibited high optical transparency and shape-memory that assisted scratch-healing properties. The total strain recovery ratio for the polymer were found to be up to 97% after thermal treatment. The crosslinked polymer network was also capable of initiating scratch recovery at ambient temperature.

Three articles are covering the field of holography. In Kinashi et al., the dynamic holographic recording in azo-carbazole doped polyester resin is investigated [10]. The dye-doped resin film exhibited a diffraction efficiency up to 0.23% and a response time of 5.9 s when illuminated with laser light. The dyeing process presented in the article is using aqueous solutions and offers some potential advantage for the fabrication of large-sized holographic devices as well as the manufacturing of photonic devices based on any polymer film containing organic dye.

In Blanche, Mahamat, and Buoye [11], the authors have measured some of the thermal properties of Bayfol HX200 photopolymer. This photopolymer is used as an holographic material in a variety of applications and it is important to understand the impact of temperature on the optical properties of the material. Authors found that the material as well as the hologram recorded within can sustain temperature up to 160 ${}^{\circ }$C. A linear coefficient of thermal expansion (CTE) of $384\times 10^{-6}\;K^{-1}$ was calculated by measuring the spectral shift of a reflection hologram depending on temperature. This shows how temperature can dramatically affect the spectral response of holograms and how these measurements can be used to predict their behavior.

Finally, Oggioni et al. are presenting a review of holography using photochromic materials and, more specifically, diarylethenes dyes [12]. This re-writable class of material shows the complex modulation of their refractive index, meaning they are suitable for both amplitude and phase holograms. In addition, they and are self developing since they do not require any post processing treatment to obtain the final hologram. A combination of a kinetic model and experimental UV-vis data made possible the development of a computational tool to predict and optimize the performances of the material. The recording of both binary and grayscales holograms are presented using either a mask approach or the use of a DMD chip for direct laser writing.

I would like to thank all the authors that participated in this Special Issue, as well as the people I interacted with at MDPI for their help with the editorial process.
